# Ultrasonic-assisted extraction, anti-biofilm activity, and mechanism of action of Ku Shen (*Sophorae Flavescentis Radix*) extracts against *Vibrio parahaemolyticus*

**DOI:** 10.3389/fmicb.2024.1379341

**Published:** 2024-03-26

**Authors:** Yanan Zhao, Siya Guo, Shuge Li, Enjun Ye, Wenfang Wang, Tong Wang, Ying Wen, Lei Guo

**Affiliations:** ^1^Jiangsu Key Laboratory of Marine Bioresources and Environment, Co-Innovation Center of Jiangsu Marine Bio-Industry Technology, Jiangsu Ocean University, Lianyungang, China; ^2^College of Kangda, Nanjing Medical University, Lianyungang, China

**Keywords:** antibacterial, antibiofilm, Ku Shen, *Vibrio parahaemolyticus*, ultrasonic-assisted extraction

## Abstract

The objective of this study is to optimize the ultrasonic-assisted extraction process of Ku Shen (*Sophorae Flavescentis Radix*) extracts (KSE) against *Vibrio parahaemolyticus* and explore their anti-biofilm activity and mechanism of action. The ultrasonic-assisted extraction process of KSE optimized by single factor experiment, Box–Behnken design and response surface methodology was as follows: 93% ethanol as solvent, liquid/material ratio of 30 mL/g, ultrasonic power of 500 W, extraction temperature of 80°C and time of 30 min. Under these conditions, the diameter of inhibition circle of KSE was 15.60 ± 0.17 mm, which had no significant difference with the predicted value. The yield of dried KSE is 32.32 ± 0.57% and the content of total flavonoids in KSE was 57.02 ± 5.54%. The minimum inhibitory concentration (MIC) and minimum bactericidal concentration (MBC) of KSE against *V. parahaemolyticus* were 0.25 and 0.5 mg/mL, respectively. Crystal violet staining, Congo red plate, spectrophotometry, CCK-8 and scanning electron microscopy were used to investigate the activity and mechanism of action of KSE against *V. parahaemolyticus* biofilm. The results showed that the sub-MIC of KSE could significantly inhibit biofilm formation, reduce the synthesis of polysaccharide intercellular adhesin (PIA) and the secretion of extracellular DNA. In addition, the inhibition rate of biofilm formation and clearance rate of mature biofilm of 1.0 mg/mL KSE were 85.32 and 74.04%, and the reduction rate of metabolic activity of developing and mature biofilm were 77.98 and 74.46%, respectively. These results were confirmed by visual images obtained by scanning electron microscopy. Therefore, KSE has the potential to further isolate the anti-biofilm agent and evaluate it for the preservation process of aquatic products.

## 1 Introduction

*Vibrio parahaemolyticus* is a halophilic bacterium commonly found in coastal seawater, seafloor sediments and seafood such as fish and shrimp (Guo et al., 2022a). With the increase of seafood consumption, *V. parahaemolyticus* poses an increasing threat to aquaculture and is one of the main pathogens leading to the mass death of aquaculture animals (Ge et al., 2019; Guo et al., 2022b). Meanwhile, *V. parahaemolyticus* is also the main cause of food poisoning. After eating raw or undercooked seafood contaminated by *V. parahaemolyticus*, consumers may suffer from diarrhea, nausea and fever, and even dehydration, shock and coma in severe cases (Mizan et al., 2018; Ashrafudoulla et al., 2019; Chen et al., 2020; Zhu et al., 2022). Furthermore, *V. parahaemolyticus* can form biofilms in the process of infection. The existence of biofilms can not only reduce the heat exchange rate of equipment, increase energy consumption, but also corrode the surface of equipment. During the production and processing of food, pathogenic bacteria are continuously released to cause continuous pollution to food, shortening the shelf life of products. Moreover, the tolerance of biofilm to antibiotics and disinfectants is tens or even hundreds of times that of planktonic bacteria, so traditional chemosynthetic antimicrobial agents have poor inhibitory effect on *V. parahaemolyticus* biofilms, and even have side effects on human body, resulting in secondary pollution (Cerca et al., 2005). In particular, the safety of synthetic antimicrobials is the focus of attention. Consumers are increasingly aware of the adverse effects of synthetic antimicrobials on the environment and health. Therefore, there is an urgent need for aquatic product preservatives with little or no chemical additives.

As the strong historical evidence of the Chinese nation, Chinese herbal medicine has been used in China and some Asian countries for thousands of years, and a variety of Chinese herbal medicine has been clinically proven to have favorable antibacterial effect (Zhao et al., 2019). Meanwhile, Chinese herbal medicine has the advantages of wide source, cheap price, multiple active ingredients, and less drug resistance, etc., which is a high-quality resource for the development of green, safe and efficient antimicrobial alternatives (Guo et al., 2017; Zheng et al., 2022).

In our preliminary screening experiment, it was found that 80% ethanol water extract of Ku Shen had a good activity against *V. parahaemolyticus* with inhibition zone of 15.05 ± 0.36 mm. Ku Shen, also called as *Sophorae Flavescentis Radix*, refers to the dried root of *Sophora flavescenes* Ait., which is widely used in combination with other medicinal plants to treat fever, dysentery, hematochezia, jaundice, oliguria, vulvar swelling, asthma, eczema, inflammatory diseases, ulcers and diseases related to skin burns (He et al., 2015). Modern studies have shown that Ku Shen mainly contains flavonoids and alkaloids, and has a variety of pharmacological activities including antibacterial, anti-tumor, antioxidant and anti-inflammatory (Lee et al., 2017; Ma et al., 2018; Li et al., 2020; Chen et al., 2021).

Compared with the traditional maceration and Soxhlet extraction methods, the ultrasonic-assisted extraction (UAE) can significantly shorten the extraction time and obtain higher yield (Xu et al., 2017). UAE has been widely used to extract bioactive components from different biological materials (Guo et al., 2014; Xu et al., 2017; Wen et al., 2018). However, to the best of our knowledge, the UAE process of the Ku Shen (*Sophorae Flavescentis Radix*) extracts (KSE) against *V. parahaemolyticus* has not been reported. Response surface methodology (RSM) uses reasonable experimental design and certain processing of experimental data to establish the functional relationship between the independent factors and the response value and obtain a regression equation. Then, the optimal process parameters are determined by analyzing the regression equation (Guo et al., 2016). RSM has been widely used in the fields of biochemistry, food and pharmacy.

The objective of this study was to optimize the ultrasonic-assisted extraction process of KSE against *V. parahaemolyticus* by single-factor experiment, Box–Behnken design (BBD) and RSM, and investigate their anti-biofilm activity and mechanism of action.

## 2 Materials and methods

### 2.1 Material and reagents

Ku Shen (*Sophorae Flavescentis Radix*) was purchased from Bozhou Traditional Chinese Medicine Factory (Anhui, China), crushed and sieved through 40 meshes for later use. *V. parahaemolyticus* 1.1997 was purchased from China General Microbiological Culture Collection Center. Crystal violet was purchased from Guangdong Huankai Microbial Technology Co., Ltd. CCK-8 cell viability test kit was purchased from Fuzhou Feijing Biotechnology Co., Ltd. The bacterial genomic DNA extraction kit was purchased from Chengdu Fuji Biotechnology Co., Ltd. Mueller-Hinton agar (MHA) and Mueller-Hinton broth (MHB) media were purchased from Hangzhou Baisi Biotechnology Co., Ltd. Congo red and other chemical reagents (analytical grade) were purchased from Sinopharm Chemical Reagent Co., Ltd.

### 2.2 Single-factor experiment

One gram of Ku Shen powder was accurately weighed and added into 250 mL conical flask. The flask was attached to the center of SK8210LHC ultrasonic extractor (40 kHz). The heating water level should exceed the conical flask’s water level, and extract under reflux condensation. The factors and levels of the single-factor experiment are shown in Table 1. After each extraction, the extract was filtered through a Buchner funnel under reduced pressure and making the volume of 30 mL for determining the activity of the extracts against *V. parahaemolyticus*.

**Table 1:** Factors, levels, and other extraction conditions for single factor experiments.

Variables	Levels	Other extraction conditions
Ethanol concentration (%)	60, 70, 80, 90, 100	L/M ratio 30 mL/g, ultrasonic power 500 W, temperature 70°C, and time 20 min
Ultrasonic power (W)	200, 300, 400, 500	L/M ratio 30 mL/g, ethanol concentration 90%, temperature 70°C, and time 20 min
Extraction temperature (°C)	40, 50, 60, 70, 80	L/M ratio 30 mL/g, ethanol concentration 90%, ultrasonic power 500 W, and time 20 min
Extraction time (min)	5, 10, 20, 30, 40	L/M ratio 30 mL/g, ethanol concentration 90%, ultrasonic power 500 W, and temperature 70°C
L/M ratio (mL/g)	5, 10, 20, 30, 40	ethanol concentration 90%, ultrasonic power 500 W, temperature 70°C, and time 20 min

### 2.3 Box–Behnken design

Box–Behnken design (BBD) was used to optimize the ultrasonic-assisted extraction conditions, and the experimental data were analyzed and the model was established using the Design Expert 8.0.0. Ethanol concentration (*X*_1_), extraction temperature (*X*_2_) and extraction time (*X*_3_) were adopted as independent variables, and the diameter of inhibitory plaque of KSE against *V. parahaemolyticus* was used as response variable (*Y*, mm). The application of RSM can provide the empirical relationships between independent variables and response variables on the basis of parameter estimation (Guo et al., 2023). The following second-order polynomial equation is used to evaluate the system mode:


$$Y=\beta_{0}+\overset{3}{\underset{i=1}{\sum }}\beta_{i}X_{i}+\overset{3}{\underset{i=1}{\sum }}\beta_{ii}X_{i}^{2}+\overset{2}{\underset{i=1}{\sum }}\overset{3}{\underset{j=i+1}{\sum }}\beta_{ij}X_{i}X_{j}$$

Where, *Y* is the predicted response value; *β*_0_, *β*_i_, *β*_ii_ and *β*_ij_ is the regression coefficient of intercept, linear and quadratic terms; *X*_i_ and *X*_j_ are independent factors.

### 2.4 Determination of antibacterial activity of the extracts

The antibacterial activity of the extracts against *V. parahaemolyticus* was determined by Oxford cup method according to the reference (Xu et al., 2021). Briefly, 200 μL of the extracts was added into the Oxford cups of MHA medium precoated with *V. parahaemolyticus*. The dishes were incubated at 32°C for 24 h, and then the diameter of the inhibition zone was determined using electronic vernier calipers. The experiments were conducted three times in parallel, and the mean value of the diameter of inhibitory plaque was recorded as the anti-*V. parahaemolyticus* activity of the extracts.

### 2.5 Determination of total flavonoids in KSE

KSE was obtained by evaporating and drying the extracts under reduced pressure. The content of total flavonoids in KSE was determined by aluminum nitrate colorimetric method (Zhou et al., 2018). Briefly, the formononetin solution (0.2 mg/mL) dissolved in 60% ethanol was used to make the standard curve. The standard solution in different volumes (0, 0.5, 1.0, 1.25, 1.5, 2.0 mL) was accurately absorbed into a 10 mL test tube, and then supplemented to 2.0 mL with 60% ethanol. 0.2 mL 5% NaNO_2_ was added and mixed well. After 6 min, 0.2 mL 10% Al(NO_3_)_3_ was added and mixed well, 6 min later, 2 mL 1.0 mol/L NaOH was added. After 15 min, the absorbance of the solution was measured at 510 nm, and the standard curve (*Y* = 1.782*X* + 0.0408, *R*^2^ = 0.9997) was drawn with the absorbance as the ordinate and the concentration of formononetin (mg/mL) as the horizontal coordinate. According to the above operation, 0.2 mg/mL KSE solution dissolved in 60% ethanol replaced the standard solution. After the reaction, the absorbance at 510 nm was determined, and the content of total flavonoids in KSE was calculated by substituting the standard curve equation.

### 2.6 Determination of minimal inhibitory concentration and minimal bactericide concentration

The minimum inhibitory concentration (MIC) of KSE against *V. parahaemolyticus* was determined by Oxford cup method according to the literature (Guo et al., 2019). Briefly, KSE was diluted into a series of solutions by the twofold dilution method, and the operation was conducted according to Oxford cup method above. The experiment was carried out twice in parallel, and the minimum concentration which both could produce the inhibition zone was recorded as the MIC value.

The MIC and minimum bactericidal concentration (MBC) of KSE against *V. parahaemolyticus* was determined by test tube method according to literature (Guo et al., 2019). Briefly, 50 μL of bacterial suspension (10^6^ cells/mL) and 50 μL of KSE solution were added into the tube (2.5 × 20.5 cm) containing 4.9 mL MHB medium. The final concentrations of KSE were 2, 1, 0.5, 0.25, 0.125, 0.0625, 0.03125 mg/mL, respectively. The tube with 50 μL of 93% ethanol instead of the sample solution was used as the negative control. The MIC was defined as the lowest concentration corresponding to the tube without bacterial growth observed by naked eye after fully shaking and incubating at 37°C for 24 h. Then, 100 μL of suspensions in tubes without bacterial growth were, respectively, spread on MHA medium, and incubated at 37°C for 24 h. MBC was defined as the minimum concentration corresponding to plates with no colonies or fewer than 5 colonies.

### 2.7 Determination of antibiofilm activity by crystal violet staining assay

The inhibitory effect of KSE on biofilm formation of *V. parahaemolyticus* and the scavenging effect of mature biofilm were determined by crystal violet staining assay (Zhang et al., 2023). For the inhibition of biofilm formation experiments, overnight cultures of *V. parahaemolyticus* were adjusted to approximately 10^6^ cells/mL and inoculated into 96-well microtiter plates (180 μL/well). Each concentration of KSE solution was added into each well at 20 μL/well to reach the final concentration of 0, 0.125, 0.25, 0.5 and 1.0 mg/mL. The culture was incubated at 37°C for 8 and 24 h, respectively; For the mature biofilm removal experiment, *V. parahaemolyticus* suspension (10^6^ cells/mL) was inoculated into a 96-well microtiter plate (180 μL/well) for 24 h to form a mature biofilm, and washed twice with PBS to remove the free bacteria. Then 180 μL fresh MHB and 20 μL KSE solutions were added into each well to make the final concentrations of 0, 1, 2, 4 and 8 mg/mL, respectively. The cultures were incubated at 37°C for 24 and 48 h, respectively.

The culture medium was discarded, washed twice with 200 μL PBS, and then dried and fixed for 30 min at room temperature. The biofilm was stained with 0.1% crystal violet (200 μL/well) for 20 min, then the staining solution was discarded, and the biofilm was washed twice with PBS (200 μL/well). After being dissolved in 200 μL/well of 33% acetic acid for 10 min, the absorbance was measured at 595 nm using a microplate reader.

### 2.8 Determination of polysaccharide intercellular adhesin by Congo red plate assay

The effect of KSE on the synthesis of polysaccharide intercellular adhesin (PIA) during biofilm formation of *V. parahaemolyticus* was determined by Congo red plate assay (Xiang et al., 2017). Single colonies of *V. parahaemolyticus* cultured overnight were inoculated onto Congo red plates containing different concentrations of KSE (0, 0.125, 0.25, 0.5 and 1 mg/mL). The results were observed after 24 and 48 h of inverted culture at 37°C, respectively.

### 2.9 Determination of extracellular DNA by spectrophotometry

The inhibitory effect of KSE on extracellular DNA (eDNA) secretion during biofilm formation of *V. parahaemolyticus* was determined by spectrophotometry (Yan et al., 2017). *V. parahaemolyticus* suspension (10^6^ cells/mL) was added into a 96-well plate (180 μL/well), and then 20 μL of KSE was added into the suspension to reach the final concentrations of 0, 0.0625, 0.125, 0.25 and 0.5 mg/mL, respectively. After culture at 37°C for 24 h, the supernatants were discarded. The unwashed biofilm was then re-suspended in 50 mM Tris·HCl/10 mM ETDA/500 mM NaCl at pH 8.0 and transferred to the centrifuge tube. After centrifugation at 4°C at 10,000 rpm/min for 10 min, the supernatant was extracted by bacterial genomic DNA extraction kit, and the OD_260_/OD_280_ ratio was detected by spectrophotometry. The relative expression of eDNA was expressed as eDNA content per unit of biofilm.

### 2.10 Determination of biofilm metabolic activity by CCK-8 assay

The effect of KSE on biofilm metabolic activity of *V. parahaemolyticus* was determined by CCK-8 assay (An et al., 2022). For the inhibition of the metabolic activity during biofilm formation, overnight cultures of *V. parahaemolyticus* were adjusted to approximately 10^6^ cells/mL and inoculated into 96-well microtiter plates (180 μL/well). Each concentration of KSE solution was added into each well at 20 μL/well to reach the final concentration of 0, 0.5, 1, 2 and 4 mg/mL, respectively; For the inhibition of the metabolic activity on the mature biofilm, *V. parahaemolyticus* suspension (10^6^ cells/mL) was inoculated into a 96-well microtiter plate (180 μL/well) for 24 h to form a mature biofilm, and washed twice with PBS to remove the free bacteria. Then 180 μL fresh MHB and 20 μL KSE solutions were added into each well to make the final concentrations of 0, 0.5, 1, 2 and 4 mg/mL, respectively. The cultures were incubated at 37°C for 24 h, respectively. The culture medium was discarded and washed twice with 200 μL PBS to remove planktonic and loosely attached cells. Each well was incubated with 180 μL PBS and 20 μL CCK-8 in the dark at 37°C for 4 h with moderate shaking, and then the absorbance was measured at 600 nm using a microplate reader.

### 2.11 Observation of biofilm cell morphology by scanning electron microscopy

The effects of KSE on the cell morphology of *V. parahaemolyticus* biofilm were observed by scanning electron microscopy (SEM) (Wang et al., 2016). After *V. parahaemolyticus* suspension (10^6^ cells/mL) on a 24-well microtiter plate (200 μL/well) was cultured at 37°C for 24 h to form a mature biofilm on a sterile round glass cover glass (φ8 mm), KSE was added to the well to reach a final concentration of 0 and 0.5 mg/mL and incubated at 37°C for 24 h. The culture medium was discarded, the glass coverslip in the well was washed twice with PBS, and the biofilm was fixed overnight with 2.5% (v/v) glutaraldehyde at 4°C, then washed with PBS and successively dehydrated with ethanol (50, 70, 90, and 100%). The biofilms were plated by sputtering under vacuum and observed using a scanning electron microscope (SU8010, Hitachi, Japan).

### 2.12 Statistical analysis

All experiments were repeated three times, and the results were expressed as mean ± standard deviation. Student’s t-test was used for statistical analysis, and there were significant differences between different letters (*p* < 0.05).

## 3 Results

### 3.1 Optimization of ultrasonic-assisted extraction process of KSE

**Figure 1:**
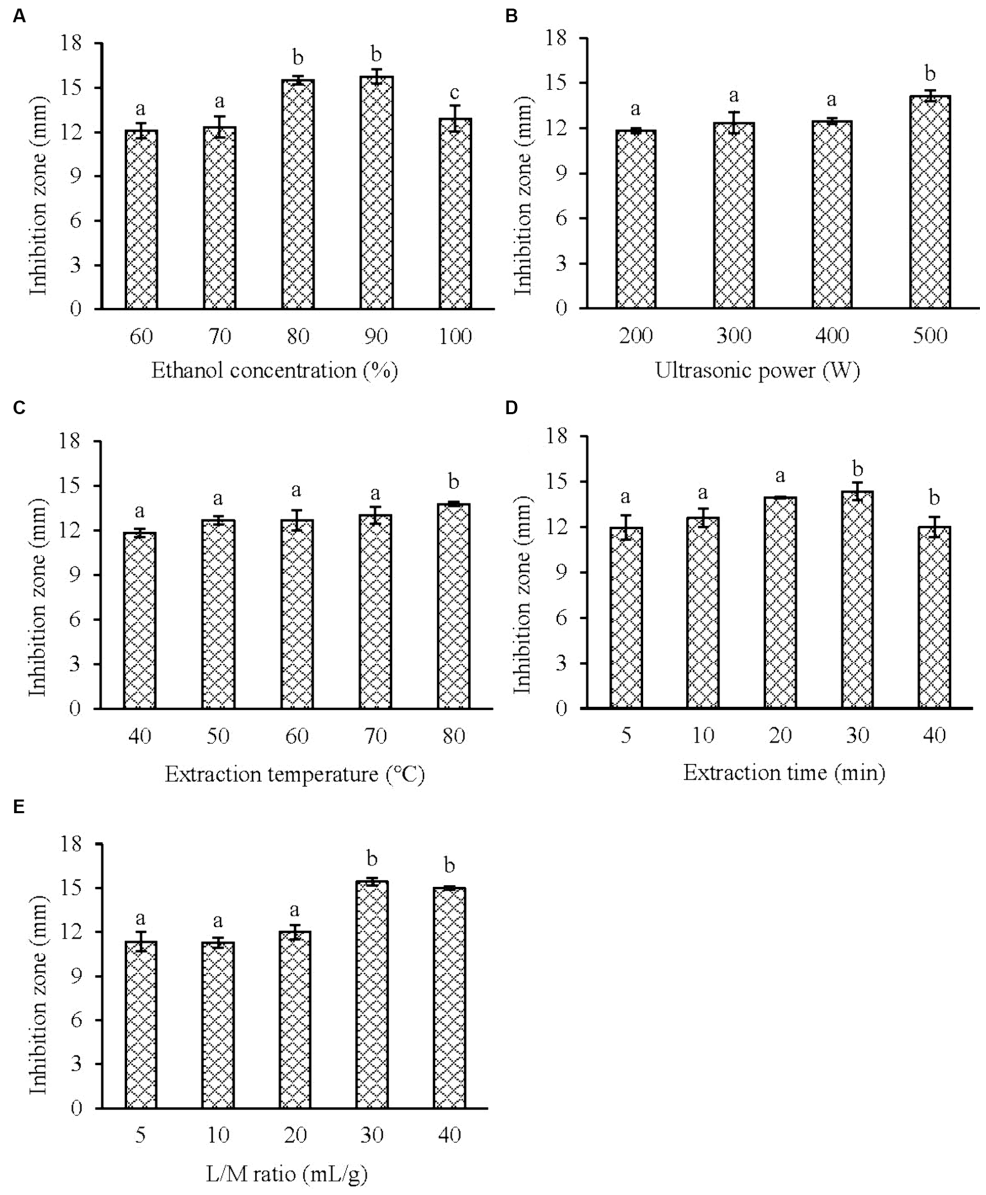
Effect of the single factor on the antibacterial activity of KSE against *V. parahaemolyticus*. **(A)** Ethanol concentration; **(B)** ultrasonic power; **(C)** extraction temperature; **(D)** extraction time and **(E)** liquid/material ratio. Compared with the previous column, different letters represent significant difference. Different lowercase letters indicated significant differences (*p* < 0.05).

Before the optimization experiment, the effects of ethanol concentration, ultrasonic power, ultrasonic temperature, ultrasonic time and liquid/material ratio on the anti-*V. parahaemolyticus* activity of KSE were investigated. Figure 1A shows that with the increasing ethanol concentration, the diameter of the inhibition zone of KSE increased, reaching the maximum at 90% ethanol (ethanol with different concentrations did not show the inhibitory halos when determined by Oxford cup method), which indicates that KSE has strong hydrophobicity. Figure 1B shows that when the ultrasonic power increased from 200 W to 500 W (100%), the diameter of the antibacterial zone of KSE increased with the increase of the power, indicating that the increase of the power increased the diffusion coefficient of the active substance and thus increased the solubility of KSE. Figure 1C shows that the diameter of the inhibition zone of KSE increased with the increase of temperature from 40°C to 80°C, indicating that the appropriate increase of temperature reduced the viscosity of the liquid, increased the diffusion coefficient, and thus increased the dissolution of the active substances. Figure 1D shows that when the extraction time was 5–30 min, the diameter of inhibitory plaque increased with time, and after 30 min, the change of the inhibitory plaque diameter was no longer significant. Figure 1E shows that when the liquid/material ratio increased from 10:1 mL/g to 30:1 mL/g, the diameter of inhibitory plaque increased accordingly. After 30:1 mL/g, the diameter of the inhibition zone did not change significantly with the increase of the liquid/material ratio.

Therefore, the ultrasonic power and liquid/material ratio were fixed at 500 W and 30 mL/g, the ethanol concentration of 90%, the ultrasonic temperature of 70°C, and the extraction time of 20 min were selected as the center points of the three independent factors in BBD to optimize the UAE process of KSE. BBD consists of seventeen test points, of which twelve are factorial points and five are replicates of central point. Ethanol concentration (*X*_1_), ultrasonic temperature (*X*_2_) and extraction time (*X*_3_) were taken as independent variables, and the diameter of inhibitory plaque of KSE was taken as response variable (*Y*, mm). The design values were shown in Table 2.

**Table 2:** The range and level of the independent variables and the observed values of Box–Behnken design.

No.	*X*_1_	*X*_2_	*X*_3_	*Y*
	Ethanol (%)	Temperature (°C)	Time (min)	Inhibition zone (mm)
1	−1 (80)	−1 (60)	0 (20)	11.85
2	+1 (100)	−1 (60)	0 (20)	14.21
3	−1 (80)	+1 (80)	0 (20)	14.43
4	+1 (100)	+1 (80)	0 (20)	14.21
5	−1 (80)	0 (70)	−1 (10)	12.71
6	+1 (100)	0 (70)	−1 (10)	14.04
7	−1 (80)	0 (70)	+1 (30)	13.06
8	+1 (100)	0 (70)	+1 (30)	15.08
9	0 (90)	−1 (60)	−1 (10)	12.70
10	0 (90)	+1 (80)	−1 (10)	14.88
11	0 (90)	−1 (60)	+1 (30)	14.11
12	0 (90)	+1 (80)	+1 (30)	16.40
13	0 (90)	0 (70)	0 (20)	14.00
14	0 (90)	0 (70)	0 (20)	13.82
15	0 (90)	0 (70)	0 (20)	13.73
16	0 (90)	0 (70)	0 (20)	12.87
17	0 (90)	0 (70)	0 (20)	13.47

Through multiple regression analysis of the experimental data, the second-order polynomial equation was established as follows:


$$\begin{array}{l} Y=13.58+0.69X_{1}+0.88X_{2}+0.54X_{3}-0.65X_{1}X_{2}+0.17X_{1}X_{3}\\ \;+0.027X_{2}X_{3}-0.35{X_{1}}^{2}+0.45{X_{2}}^{2}+0.5{X_{3}}^{2} \end{array}$$

Analysis of variance was conducted on the experimental data in Table 2, and the results were shown in Table 3. As can be seen from Table 3, the *p* value of the regression model is very significant (*p* = 0.0072 < 0.01), while the value of lack-of-fit is not significant (*p* = 0.3229 > 0.05), indicating that the model has a good degree of fit and is statistically significant. The regression coefficient *R*^2^ = 0.9065, close to 1, indicating that the model has a good correlation and can be used to predict the results. The coefficient of variation (C.V.) of the model was 3.55%, indicating that the model had high precision and reliability. The influence of ethanol concentration and extraction temperature is very significant (*p* < 0.01), and the influence of extraction time, the interaction of ethanol concentration and extraction temperature is significant (*p* < 0.05), which indicates that the relationship between each factor and the response value is not a simple linear relationship. The effects of different factors on the antibacterial activity of KSE against *V. parahaemolyticus* were as follows: extraction temperature > ethanol concentration > extraction time.

**Table 3:** Variance analysis table.

Source	Sum of squares	df	Mean square	*F* value	Prob > F	Sig.
Model	16.46	9	1.83	7.54	0.0072	**
*X*_1_	3.77	1	3.77	15.53	0.0056	**
*X*_2_	6.21	1	6.21	25.60	0.0015	**
*X*_3_	2.33	1	2.33	9.61	0.0173	*
*X*_1_*X*_2_	1.66	1	1.66	6.86	0.0345	*
*X*_1_*X*_3_	0.12	1	0.12	0.49	0.5063	
*X*_2_*X*_3_	0.003	1	0.003	0.01	0.9142	
*X*_1_^2^	0.52	1	0.52	2.14	0.1866	
*X*_2_^2^	0.85	1	0.85	3.49	0.1039	
*X*_3_^2^	1.04	1	1.04	4.27	0.0777	
Lack of fit	0.93	3	0.31	1.60	0.3229	

*p < 0.05, **p < 0.01.

**Figure 2:**
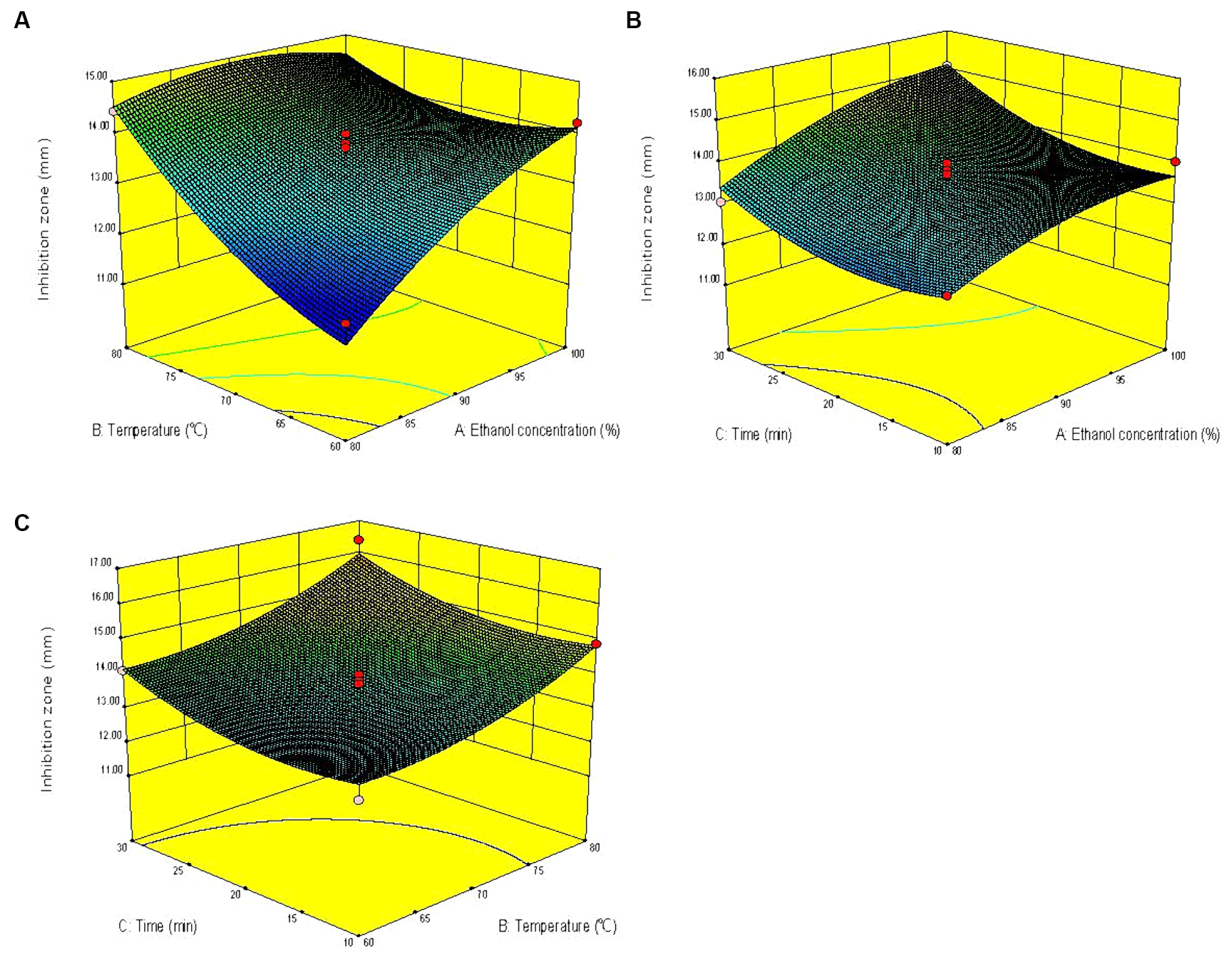
Response surface diagrams showing effects of pairwise variables on the anti-*V. parahaemolyticus* activity of KSE and their interaction. **(A)** Ethanol concentration and temperature; **(B)** ethanol concentration and time; **(C)** temperature and time.

The three-dimensional response surface graph intuitively reflects the influence of one variable fixed at the central level and the other two variables on the anti-*V. parahaemolyticus* activity of KSE. In the response surface diagram, the steeper the slope of the response surface is, the more sensitive the response value is. Conversely, the slower the slope is, the less obvious the influence of this variable on the response value is (Guo et al., 2016). According to the slope of the response surface in Figure 2, the curve trend of ethanol concentration and extraction temperature was steep, indicating that the interaction of ethanol concentration and extraction temperature had a significant effect on the anti-*V. parahaemolyticus* activity of KSE, while the interaction of ethanol concentration and extraction time, extraction temperature and time had no significant effect.

The optimal extraction conditions predicted by the model were *X*_1_ = 93%, *X*_2_ = 80°C, *X*_3_ = 30 min, and the predicted value of antibacterial zone diameter was 16.00 mm. The validation experiment was conducted under the above optimized conditions, and the actual mean value of the antibacterial zone diameter was 15.60 ± 0.17 mm, which had no significant difference from the predicted value. The results showed that the combination of single-factor experiment, BBD and RSM had good feasibility to optimize the UAE process of KSE against *V. parahaemolyticus*.

The yield of dried KSE is 32.32 ± 0.57% and the content of total flavonoids in KSE was 57.02 ± 5.54%. Subsequently, the dried flavonoid-rich KSE were used to further investigate the anti-biofilm activity and their mechanism of action against *V. parahaemolyticus*.

### 3.2 Antibacterial and antibiofilm activities of KSE against *Vibrio parahaemolyticus*

The MIC of KSE against *V. parahaemolyticus* was determined by Oxford cup method and test-tube method, and the results were consistent, both of which were 0.25 mg/mL. Further, the MBC of KSE against *V. parahaemolyticus* measured by the tube dilution combined with plates coating method was 0.5 mg/mL.

**Figure 3:**
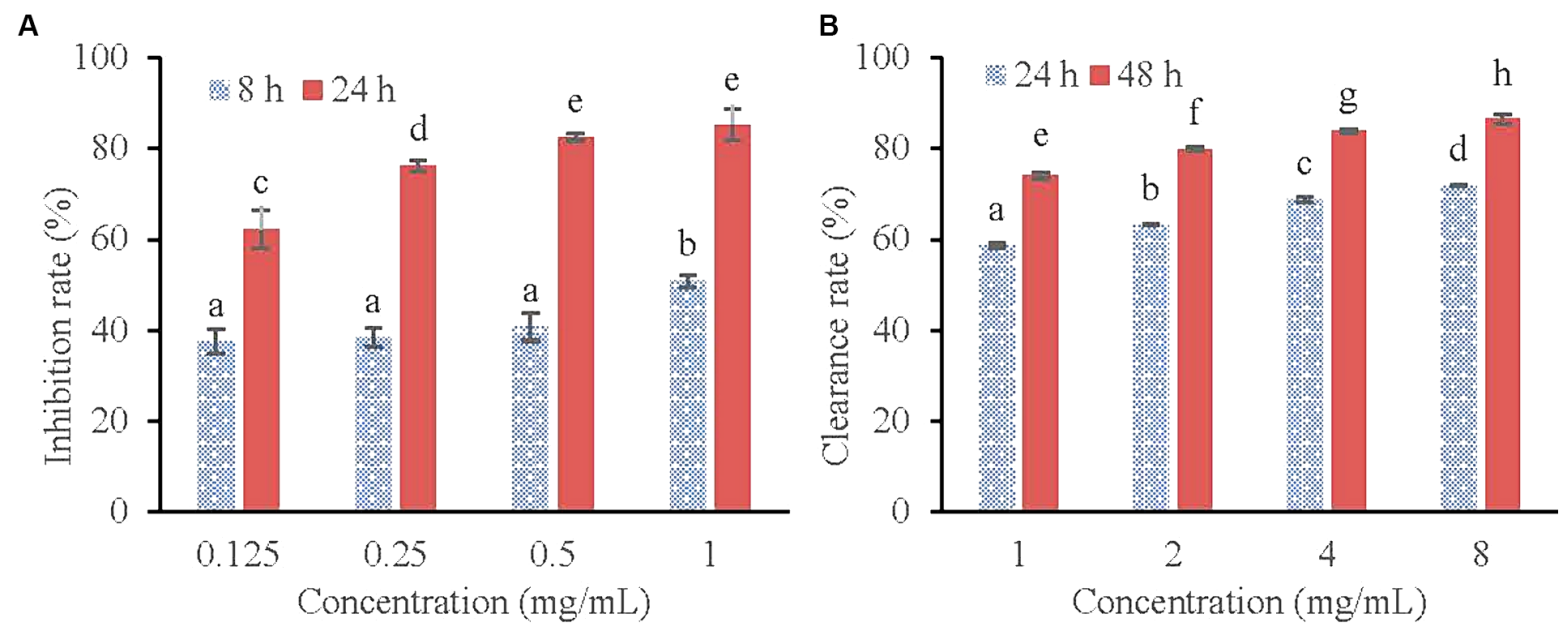
Inhibitory effect of KSE on the biofilm formation **(A)** and Clearing effect of KSE on the mature biofilm **(B)** of *V. parahaemolyticus*. Different lowercase letters indicated significant differences (*p* < 0.05).

The inhibitory effect of KSE on the biofilm formation of *V. parahaemolyticus* is shown in Figure 3A. As can be seen from Figure 3A, the inhibitory effect of KSE on the biofilm formation of *V. parahaemolyticus* increased with the extension of time (*p* < 0.05). The inhibitory rates of 0.125, 0.25, 0.5 and 1 mg/mL KSE on biofilm formation increased from 37.60, 38.37, 40.70 and 50.78% at 8 h to 62.26, 76.29, 82.58, and 85.32% at 24 h, respectively, showing a certain concentration dependence.

Figure 3B shows the scavenging effect of KSE on mature biofilm of *V. parahaemolyticus*. It can be seen that the scavenging effect of KSE on the mature biofilm of *V. parahaemolyticus* increased with the extension of the treatment time. The clearance rates of 1, 2, 4 and 8 mg/mL KSE on mature biofilm increased from 58.59, 63.15, 68.70, and 71.90% at 24 h to 74.04, 79.92, 83.90, and 86.56% at 48 h, respectively. Both time and concentration dependence were observed (*p* < 0.05).

### 3.3 Antibiofilm mechanisms of KSE against *Vibrio parahaemolyticus*

**Figure 4:**
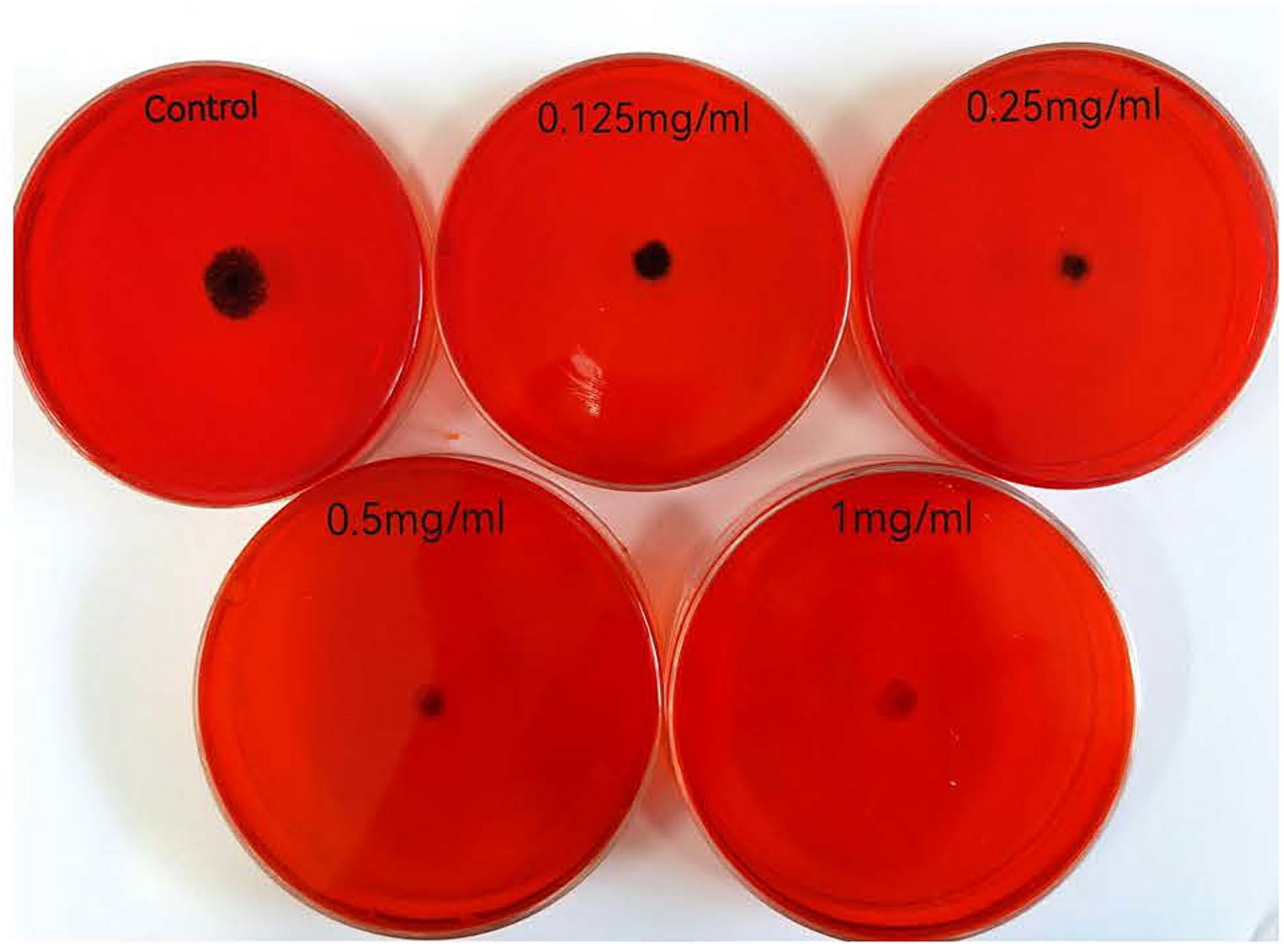
Effect of KSE on PIA synthesis of *V. parahaemolyticus*.

After *V. parahaemolyticus* was inoculated into Congo red plate and cultured, the black colonies were PIA-positive colonies, while the red colonies were PIA negative colonies. As can be seen from Figure 4, with the increase of the concentration of KSE, the amount of PIA synthesis gradually decreased, indicating that KSE can significantly inhibit the secretion of PIA during the biofilm formation of *V. parahaemolyticus*.

**Figure 5:**
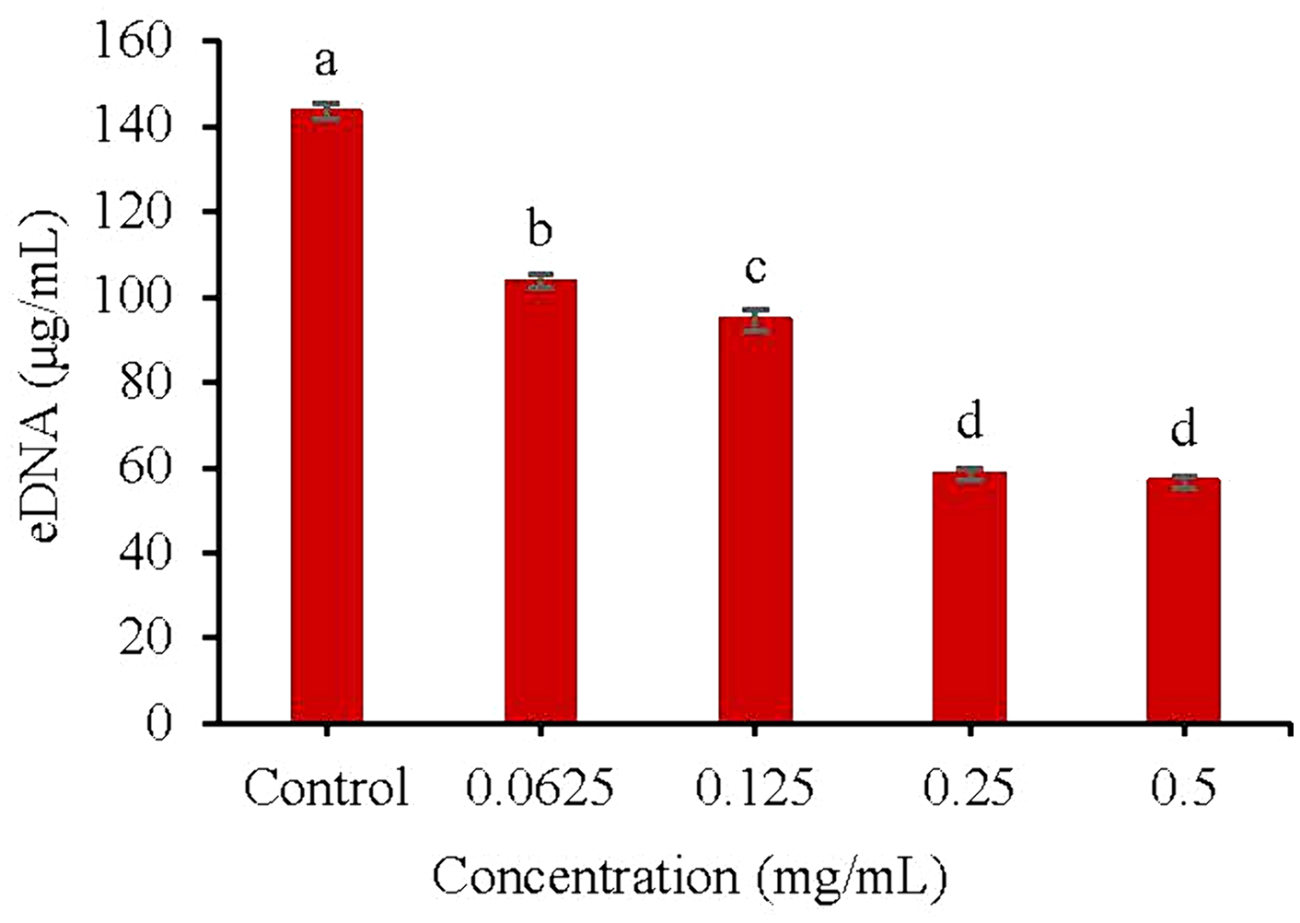
Effect of KSE on eDNA secretion of *V. parahaemolyticus*. Different lowercase letters indicated significant differences (*p* < 0.05).

As shown in Figure 5, a low concentration (0.0625 mg/mL) of KSE could significantly inhibit extracellular DNA (eDNA) secretion during the biofilm formation of *V. parahaemolyticus*. With the increasing concentration of KSE, the inhibitory effect was strengthened. Compared with the control group, 0.25 mg/mL KSE could reduce the eDNA secretion from 143.3 to 58.40 μg/mL, that is, the eDNA secretion was reduced by 59.25% (*p* < 0.01).

**Figure 6:**
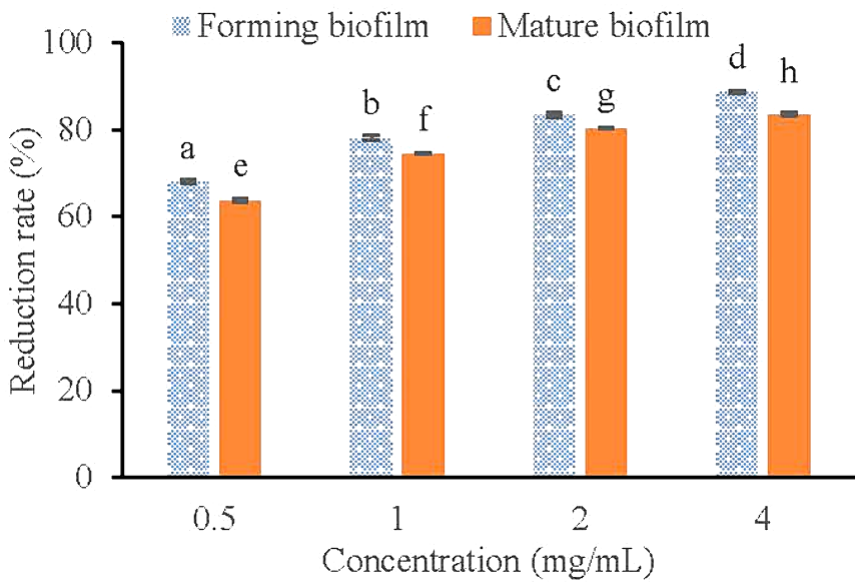
Effect of KSE on biofilm metabolic activity of *V. parahaemolyticus*. Different lowercase letters indicated significant differences (*p* < 0.05).

The CCK-8 cell motility assay kit contains the reagent WST-8, which is reduced to a highly water-soluble yellow formazan dye by the cell dehydrogenase under the action of the electron carrier 1-methoxy-5-methylphenazine-dimethyl ester sulfate (1-Methoxy PMS). Its color depth is directly proportional to cell metabolic activity (An et al., 2022). As shown in Figure 6, Compared with the control group, 0.5, 1.0, 2.0, and 4.0 mg/mL of KSE reduced the metabolic activity of *V. parahaemolyticus* during the biofilm formation by 67.97, 77.98, 83.31, and 88.70%, respectively, which was higher than that of mature biofilm, that is 63.71, 74.46, 80.21, and 83.39%, respectively. The results showed that KSE could significantly reduce the metabolic activity of cells in forming and matured *V. parahaemolyticus* biofilms in a dose-dependent manner (*p* < 0.01).

**Figure 7:**
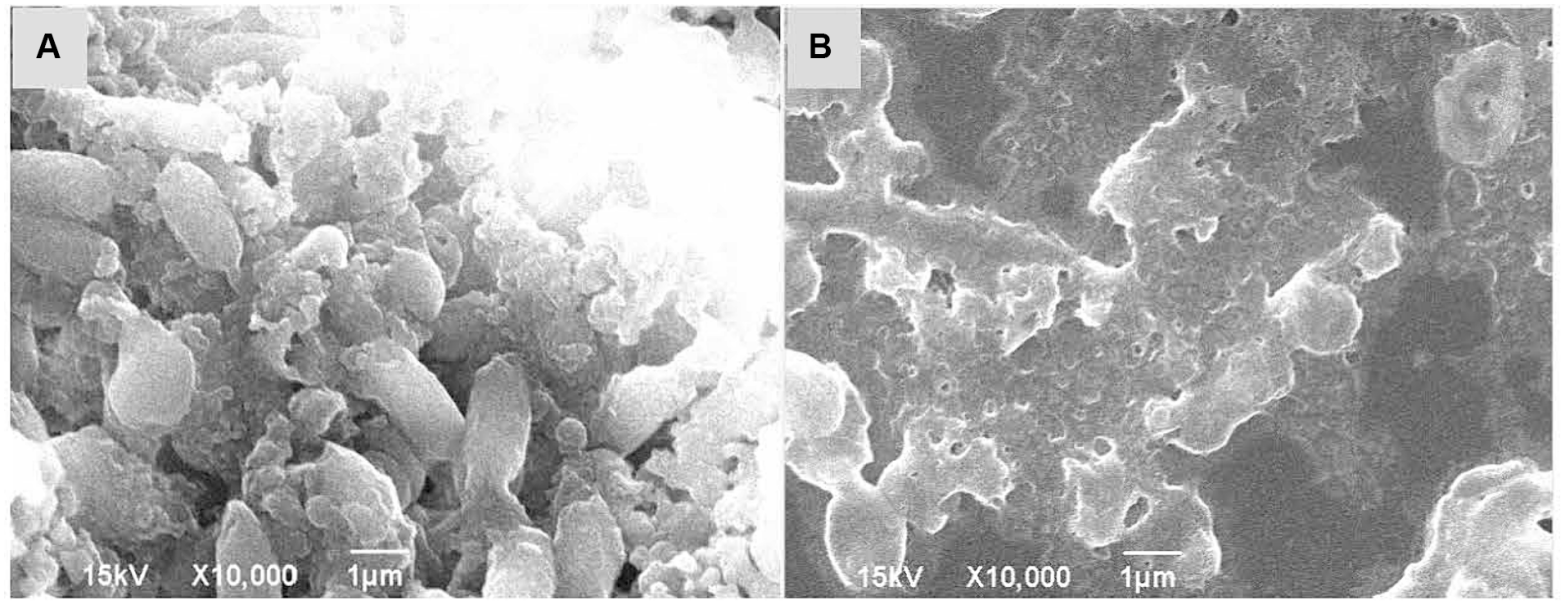
SEM analysis of *V. parahaemolyticus* without treatment **(A)** and treatment with 0.5 mg/mL KSE **(B)**.

Furthermore, SEM images showed that *V. parahaemolyticus*, which was not treated with KSE, had a short rod-like shape with an oval end and a complete morphological structure (Figure 7A). After being treated with 0.5 mg/mL KSE for 24 h, cell morphology changed, the bacteria appeared serious distortion, protoplasm leakage, and cell aggregation and overlap (Figure 7B), indicating that KSE could obviously destroy the structure of *V. parahaemolyticus* biofilm cells.

## 4 Discussion

*Vibrio parahaemolyticus* is an important pathogen of seafood origin, which poses a serious threat to public health (Guo et al., 2022b). Due to the increasing incidence of foodborne diseases caused by this pathogen, new strategies against *V. parahaemolyticus* are urgently needed. Chinese herbal medicine is an excellent source for finding new antimicrobial agents due to its long history of application and rich experience used in Asia over the past thousands of years. Ren et al. screened the anti-*V. parahaemolyticus* activities of 38 kinds of Chinese herbal medicine, and found that Chinese gallnut, pomegranate peel, *Baikal skullcap* root and Coptidis rhizoma had the strongest bacteriostasis and bactericidal abilities, with MIC and MBC of 1.56 mg/mL and 3.13 mg/mL, respectively (Ren et al., 2020). The favorable inhibitory effect of Chinese gallnut and pomegranate peel extract on *V. parahaemolyticus* has been confirmed by other studies, with MIC range of 0.04 ~ 5 mg/mL (Wu et al., 2018). Although the extract of Ku Shen has been shown to have antibacterial activities against bacteria or fungi such as *Staphylococcus aureus*, the MIC value is 0.28 mg/mL (He et al., 2015), but their anti-*V. parahaemolyticus* activity have not been reported.

UAE is considered as one of the green food processing technologies, and several parameters can be controlled to improve the efficiency of it, including several process variables that need to be considered separately (Chemat et al., 2017). The main reason why ultrasonic treatment improves the extraction efficiency is that the strong cavitation, mechanical and thermal effects significantly promote the mass transfer between immiscible phases (Wen et al., 2018). Therefore, the main variables must be identified prior to the optimization process and their responses must be maximized taking into account minimum time, energy and solvent consumption (Dai and Mumper, 2010). Single-factor experimentation is the most common form of performing optimization, where the effect of each variable is measured independently while all other variables are fixed. However, RSM is a very useful strategy that allows all variables to be optimized simultaneously and predicts the most efficient conditions. In this study, the key factors of UAE of KSE against *V. parahaemolyticus* were screened based on the single-factor experiment method, and then RSM was used to optimize the optimal extraction conditions. The actual inhibition zone of KSE (15.60 ± 0.17 mm) was not significantly different from the predicted value (16.00 mm), indicating the feasibility of the combination of single-factor experiment, BBD and RSM to optimize the UAE process of KSE against *V. parahaemolyticus*.

Total alkaloids, total flavonoids and mixtures of these compounds from *Sophora flavescens* potently inhibited *Staphylococcus aureus*, *Escherichia coli*, *Shigella flexneri*, *Bacillus paratyphosus B*, *Pseudomonas aeruginosa*, *Staphylococcus albus*, *Shigella Castellani* and *Staphylococcus citreus in vitro* with MIC values of 9.0, 3.38, 8.25, 9.0, 4.50, 13.50, 6.75, 7.13 mg/mL, 3.0, 2.63, 356, 3.38, 1.50, 2.44, 3.56, 2.35 mg/mL and 2.25, 2.07, 3.28, 3.94, 1.22, 2.44, 2.07, 1.97 mg/mL, respectively (Du et al., 2010). The results indicated that total flavonoids are the main antibacterial molecular groups of Ku Shen. Therefore, in this study, the total flavonoid content in KSE was quantified by colorimetry, i.e., 57.02 ± 5.54%, which indicated that the total flavonoids accounted for a large proportion of KSE. It is necessary to further isolate and characterize the active molecules of KSE against *V. parahaemolyticus*.

*Vibrio parahaemolyticus* biofilm is another form of existence different from its planktonic cells. Once the biofilm is formed, the number of bacteria will increase significantly, which is a natural barrier for the body to defend against and limit the access of antibacterial agents to bacteria (Abebe, 2020; Hu et al., 2022). As one of the effective strategies to find efficient and safe alternatives to chemosynthetic antimicrobials, the discovery of anti-biofilm agents has attracted wide attention in recent years (Wei et al., 2017; Lu et al., 2021). Based on this, the anti-biofilm activity and mechanism of action of KSE against *V. parahaemolyticus* were furthermore investigated in this study.

The bacteria in the biofilm are much less sensitive to drugs, and the use of antibiotics and disinfectants to clear the biofilm requires a much higher dose than that of planktonic counterparts (Cerca et al., 2005; Yan et al., 2017). Similarly, this study found that 8 to 32 times the concentration of KSE was required for remove mature biofilm to achieve equivalent activity for the inhibition on forming biofilm.

The formation of biofilm is a very complicated process, which is the result of various factors. Extracellular polysaccharide, protein and eDNA are the main components of biofilm. The synthesis of PIA plays a crucial role in the adhesion and aggregation stage of biofilm (Arciola et al., 2015). The decrease of PIA synthesis will lead to the decrease of adhesion ability between strains and the subsequent decrease of biofilm formation ability. Aloe emodin was found to reduce the accumulation of PIA on the surface of *S. aureus* biofilm (Xiang et al., 2017). eDNA is the DNA released outside the cell after the programmed death and dissolution of the strain, and play a bonding role in the adhesion stage and a role in stabilizing the structure of the mature biofilm (Das et al., 2010). Emodin with sub-MIC concentration could significantly interfere with the release of eDNA from *S. aureus* biofilm (Yan et al., 2017). The results of this study indicated that the sub-MIC concentration of KSE could significantly inhibit the PIA synthesis and eDNA release of *V. parahaemolyticus*, thus reducing the biofilm formation. It was also found that 2-fold and 4-fold MIC concentrations, namely 0.5 and 1.0 mg/mL of KSE, could significantly reduce the metabolic activity of *V. parahaemolyticus* biofilm, with a reduction rate of 63.71 and 74.46%, respectively. 4MIC (0.6%) of eugenol was found to reduce the metabolic activity of *V. parahaemolyticus* NIFS29 biofilm by 68% (Ashrafudoulla et al., 2020). Similar studies found that 4MIC (5.0 mg/mL) of chitosan reduced the metabolic activity of *V. parahaemolyticus* ATCC17802 biofilm by 76.10% (Xie et al., 2017). SEM further observed that 0.5 mg/mL KSE significantly damaged the structure of *V. parahaemolyticus* biofilm.

Based on the green origin and excellent anti-biofilm properties of KSE, the anti-biofilm active compounds can be purified and identified by chromatographic separation technology, and then the toxicity of anti-biofilm agent and the practical application potential for aquatic product preservation can be evaluated.

## 5 Conclusion

The optimized UAE conditions of KSE against *V. parahaemolyticus* were as follows: 93% ethanol as solvent, liquid/material ratio of 30 mL/g, ultrasonic power of 500 W, extraction temperature of 80°C and time of 30 min. The yield of solid KSE is 32.32 ± 0.57% and the content of total flavonoids in KSE was 57.02 ± 5.54%. The MIC and MBC of flavonoid-rich KSE against *V. parahaemolyticus* were 0.25 mg/mL and 0.5 mg/mL, respectively. In addition, KSE can significantly inhibit biofilm formation and remove mature biofilm by reducing the synthesis of PIA, the release of eDNA, the metabolic activity of biofilm and changing the bacterial structure. The results of the study provide a basis for further isolate the anti-biofilm agents in KSE and deepening their potential used in aquatic product preservation.

## Data availability statement

The original contributions presented in the study are included in the article/supplementary material, further inquiries can be directed to the corresponding author.

## Author contributions

YZ: Conceptualization, Writing – original draft. SG: Data curation, Methodology, Writing – original draft. SL: Formal analysis, Software, Writing – original draft. EY: Validation, Writing – original draft. WW: Formal analysis, Writing – original draft. TW: Formal analysis, Validation, Writing – original draft. YW: Writing – review & editing. LG: Writing – review & editing.
